# Measles Outbreak — Minnesota April–May 2017

**DOI:** 10.15585/mmwr.mm6627a1

**Published:** 2017-07-14

**Authors:** Victoria Hall, Emily Banerjee, Cynthia Kenyon, Anna Strain, Jayne Griffith, Kathryn Como-Sabetti, Jennifer Heath, Lynn Bahta, Karen Martin, Melissa McMahon, Dave Johnson, Margaret Roddy, Denise Dunn, Kristen Ehresmann

**Affiliations:** ^1^Epidemic Intelligence Service, CDC; ^2^Minnesota Department of Health; ^3^Hennepin County Human Services and Public Health Department, Minneapolis, Minnesota.

On April 10, 2017, the Minnesota Department of Health (MDH) was notified about a suspected measles case. The patient was a hospitalized child aged 25 months who was evaluated for fever and rash, with onset on April 8. The child had no history of receipt of measles-mumps-rubella (MMR) vaccine and no travel history or known exposure to measles. On April 11, MDH received a report of a second hospitalized, unvaccinated child, aged 34 months, with an acute febrile rash illness with onset on April 10. The second patient’s sibling, aged 19 months, who had also not received MMR vaccine, had similar symptoms, with rash onset on March 30. Real-time reverse transcription–polymerase chain reaction (rRT-PCR) testing of nasopharyngeal swab or throat specimens performed at MDH confirmed measles in the first two patients on April 11, and in the third patient on April 13; subsequent genotyping identified genotype B3 virus in all three patients, who attended the same child care center. MDH instituted outbreak investigation and response activities in collaboration with local health departments, health care facilities, child care facilities, and schools in affected settings. Because the outbreak occurred in a community with low MMR vaccination coverage, measles spread rapidly, resulting in thousands of exposures in child care centers, schools, and health care facilities. By May 31, 2017, a total of 65 confirmed measles cases had been reported to MDH (Figure 1); transmission is ongoing.

**FIGURE 1:**
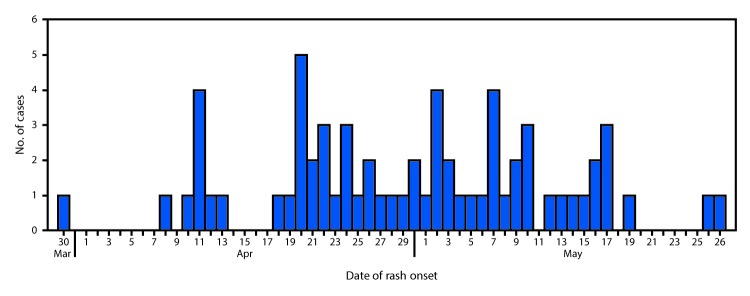
Number of measles cases (N = 65) by date of rash onset — Minnesota, March 30–May 27, 2017

## Investigation and Results

After receiving notification of the first case on April 10, MDH and the Hennepin County Human Services and Public Health Department began an investigation. The Council of State and Territorial Epidemiologists and CDC case definition* was used to identify confirmed cases of measles in Minnesota (*1*). A health alert was issued April 12, which notified health care providers of the two measles cases in Hennepin County and provided recommendations concerning laboratory testing for measles and strategies to minimize transmission in health care settings. Emphasis was placed on recommendations for all children aged ≥12 months to receive a first dose of MMR. Providers identified patients with suspected measles based on clinical findings and reported suspected cases to MDH. Testing with rRT-PCR was performed at MDH on nasopharyngeal or throat swabs and urine specimens. Among persons testing positive by rRT-PCR who had received vaccine ≤21 days before the test, genotyping was performed to distinguish wild-type measles virus (genotype B3 virus) from the vaccine virus (genotype A virus). Patients (or their parents or guardians) with confirmed measles were interviewed by local public health officials to confirm symptoms, onset date, and exposure history for the 21 days before rash onset and identify contacts during their infectious period (4 days before through 4 days after rash onset). Contacts were defined as persons who had any contact with patients during their infectious period.

Among the 65 confirmed cases, the median patient age was 21 months (range = 3 months–49 years). Patients were residents of Hennepin, Ramsey, LeSueur, and Crow Wing counties. During April 10–May 31, confirmed measles patients were identified in five schools, 12 child care centers, three health care facilities, and numerous households; an estimated 8,250 persons were potentially exposed to measles in these settings. Rash onset dates ranged from March 30–May 27, 2017. Sixty-two (95%) cases were identified in unvaccinated persons, including 50 (77%) in children aged ≥12 months (i.e., age-eligible for MMR vaccination). U.S.-born children of Somali descent (Somali children) accounted for 55 (85%) of the cases. Among the three patients with a history of measles vaccination, all had received 2 MMR doses before illness onset. As of May 31, 20 (31%) patients had been hospitalized, primarily for treatment of dehydration or pneumonia; no deaths had been reported.

## Public Health Response

Rosters and attendance records were obtained from child care centers and schools where persons might have been exposed to measles, and the vaccination status of each attendee was verified through the Minnesota Immunization Information Connection, a system that stores electronic immunization records (http://www.health.state.mn.us/miic). Health care facilities similarly identified contacts who were exposed to measles patients and followed up with susceptible (i.e., unvaccinated, pregnant, or immunocompromised) exposed persons. In accordance with the Advisory Committee on Immunization Practices 2013 guidelines (*2*), postexposure prophylaxis (PEP) with MMR or immune globulin was recommended for susceptible, exposed persons. Persons who received PEP with MMR within 72 hours of exposure or with immune globulin within 6 days of exposure were placed on a 21-day self-monitoring symptom watch for development of fever or rash, but could continue attending child care and school. Susceptible exposed persons who did not receive PEP according to recommendations were excluded from child care centers or school, and MDH recommended that they avoid public gatherings for 21 days, including having visitors who were susceptible to measles virus. By May 31, at least 154 persons had received PEP (26 MMR doses and 128 courses of immune globulin), and 586 susceptible exposed persons who did not receive recommended PEP were excluded from child care centers or school and advised to receive MMR vaccination to protect against future measles illness.

**FIGURE 2:**
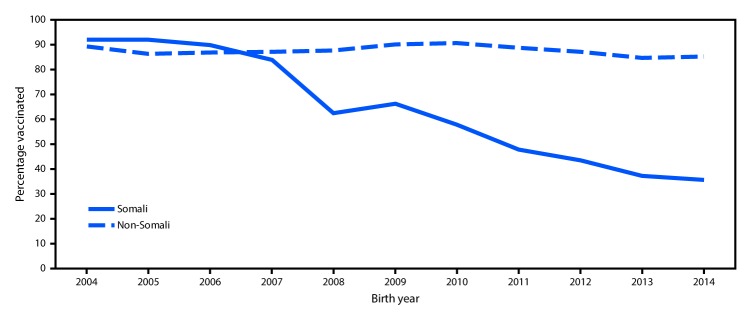
Percentage of children receiving measles-mumps-rubella vaccine at age 24 months among children of Somali and non-Somali descent, by birth year — Hennepin County, Minnesota, 2004–2014 **Source:** Minnesota Immunization Information Connection, Minnesota Department of Health.

On April 18, as the outbreak continued, MDH recommended an accelerated MMR schedule; to provide additional protection, a second dose of MMR vaccine was recommended for children who had received a first dose >28 days previously.^†^ These recommendations were initially for all children living in Hennepin County and for all Minnesota Somali children regardless of county of residence, because MMR coverage rates among Somali children in Hennepin County have declined since 2007. In 2014, coverage with the first dose of MMR among Somali children in Hennepin County was 35.6% (Figure 2). In response to the rapid increase in the number of reported cases, on May 4, 2017, MDH recommended an accelerated vaccination schedule for all children aged ≥12 months residing in all counties where a measles case had been reported during the previous 42 days; MDH further recommended that health care providers throughout the state consider using an accelerated schedule.

Previously established culturally appropriate community outreach approaches (e.g., working with community and spiritual leaders, interpreters, health care providers, and community members) (*3*) were intensified during the outbreak. Using existing partnerships, state and local public health officials worked with MDH Somali public health advisors, Somali medical professionals, faith leaders, elected officials, and other community leaders to disseminate educational materials, attend community events, and create opportunities for open dialogue and education about measles and concerns about MMR vaccine. Child care centers and schools were provided talking points and informational sheets on measles and MMR vaccine, and posters with key messages were distributed in mosques and shopping malls popular with the Somali community. Community outreach focused on oral communication, which is preferred by this community, including radio and television messaging and telephone call-in lines that permit approximately 500 persons at a time to listen to a health professional.

Outreach to encourage vaccination was increased during the outbreak. By the second week of May, the average number of MMR vaccine doses administered per week in Minnesota had increased from 2,700 doses before the outbreak to 9,964, as reported by the Minnesota Immunization Information Connection.

## Discussion

Minnesota law requires that children aged ≥2 months be vaccinated against certain diseases or file a medical or conscientious exemption to enroll in school, child care, or school-based early childhood programs. Before 2008, first-dose MMR vaccination coverage among Minnesota-born Somali children aged 2 years in Hennepin County exceeded 90%. However, MMR vaccination coverage rates declined among Minnesota’s Somali-American community members starting with the 2008 birth-year cohort. The decline in vaccination coverage was in response to concerns about autism, the perceived increased rates of autism in the Somali-American community, and the misunderstanding that autism was related to MMR vaccine (*3,4*). Studies have consistently documented that there is not a relationship between vaccines and autism (*5,6*). The low vaccination rate resulted in a community highly susceptible to measles. Parental concerns were addressed by building trust with the community and identifying effective, culturally appropriate ways to address questions, concerns, and misinformation about MMR vaccine. In 2011, a smaller measles outbreak began in the Somali community in Hennepin County and resulted in 21 cases, including eight cases in persons of Somali descent (*4*,*7*). At that time, the 1-dose MMR vaccination coverage rate among Somali children aged 2 years in Hennepin County was 54%. The source of the 2011 outbreak was a Somali child aged 30 months who acquired measles while visiting Kenya (*7*). However, the source of the current outbreak is unknown, which suggests that additional cases have likely occurred that did not come to the attention of health care providers or public health departments.

Although indigenous measles transmission has been eliminated in the United States, the virus continues to circulate widely in many regions of the world, including Africa, Europe, and parts of Asia, and is often introduced into the United States by international travelers (*8*). High measles vaccination coverage rates across subpopulations within communities are necessary to prevent the spread of measles. The current Minnesota measles outbreak, with 31% (20 of 65) of cases requiring hospitalization, demonstrates the importance of addressing low vaccination coverage rates to ensure that children are adequately protected from a potentially serious vaccine-preventable disease (*3*).

**Summary**

**What is already known about this topic?**

Measles was declared eliminated from the United States in 2000 but continues to circulate in many regions of the world and can be imported into the United States by travelers. Measles vaccine is highly effective, with 1 dose being 93% effective and 2 doses being 97% effective at preventing measles.

**What is added by this report?**

In a community with previously high vaccination coverage, concerns about autism, the perceived increased rates of autism in the Somali-American community, and the misunderstanding that autism was related to the measles-mumps-rubella (MMR) vaccine resulted in a decline in MMR vaccination coverage to a level low enough to sustain widespread measles transmission in the Somali-American community following introduction of the virus. Studies have consistently documented that there is not a relationship between vaccines and autism.

**What are the implications for public health practice?**

This outbreak demonstrates the challenge of combating misinformation about MMR vaccine and the importance of creating long-term, trusted relationships with communities to disseminate scientific information in a culturally appropriate and effective manner.

## Footnotes

* An acute illness in a Minnesota resident during January 1, 2017–May 12, 2017, characterized by generalized, maculopapular rash lasting ≥3 days with a temperature ≥101°F (≥38.3°C) and cough, coryza, or conjunctivitis. A confirmed case is an acute febrile rash illness with isolation of measles virus from a clinical specimen; or detection of measles-virus specific nucleic acid from a clinical specimen using polymerase chain reaction; or immunoglobulin G seroconversion or a significant rise in measles immunoglobulin G antibody using an evaluated and validated method; or a positive serologic test for measles immunoglobulin M antibody; or direct epidemiologic linkage to a case confirmed by one of these methods.

† The Advisory Committee on Immunization Practices (ACIP) recommends MMR vaccine for prevention of measles, mumps, and rubella for persons aged ≥12 months. ACIP recommends 2 doses of MMR vaccine routinely for children, with the first dose administered at age 12 through 15 months and the second dose administered at age 4 through 6 years before school entry. https://www.cdc.gov/mmwr/preview/mmwrhtml/rr6204a1.htm.
